# Identification of occupations susceptible to high exposure and risk associated with multiple toxicants in an observational study: National Health and Nutrition Examination Survey 1999–2014

**DOI:** 10.1093/exposome/osac004

**Published:** 2022-06-25

**Authors:** Vy Kim Nguyen, Justin Colacino, Chirag J Patel, Maureen Sartor, Olivier Jolliet

**Affiliations:** Department of Environmental Health Sciences, School of Public Health, University of Michigan, Ann Arbor, MI, USA; Department of Biomedical Informatics, Medical School, Harvard University, Boston, MA, USA; Department of Environmental Health Sciences, School of Public Health, University of Michigan, Ann Arbor, MI, USA; Department of Nutritional Sciences, School of Public Health, University of Michigan, Ann Arbor, MI, USA; Department of Biomedical Informatics, Medical School, Harvard University, Boston, MA, USA; Department of Computational Medicine and Bioinformatics, Medical School, University of Michigan, Ann Arbor, MI, USA; Department of Environmental Health Sciences, School of Public Health, University of Michigan, Ann Arbor, MI, USA; Quantitative Sustainability Assessment, Department of Environmental and Resource Engineering, Technical University of Denmark, Kgs. Lyngby, Denmark

**Keywords:** occupational epidemiology, environmental chemicals, occupational exposures, unsupervised learning, biomonitoring equivalents, risk assessment

## Abstract

Occupational exposures to toxicants are estimated to cause over 370 000 premature deaths annually. The risks due to multiple workplace chemical exposures and those occupations most susceptible to the resulting health effects remain poorly characterized. The aim of this study is to identify occupations with elevated toxicant biomarker concentrations and increased health risk associated with toxicant exposures in a diverse working US population. For this observational study of 51 008 participants, we used data from the 1999–2014 National Health and Nutrition Examination Survey. We characterized differences in chemical exposures by occupational group for 131 chemicals by applying a series of generalized linear models with the outcome as biomarker concentrations and the main predictor as the occupational groups, adjusting for age, sex, race/ethnicity, poverty income ratio, study period, and biomarker of tobacco use. For each occupational group, we calculated percentages of participants with chemical biomarker levels exceeding acceptable health-based guidelines. Blue-collar workers from “Construction,” “Professional, Scientific, Technical Services,” “Real Estate, Rental, Leasing,” “Manufacturing,” and “Wholesale Trade” have higher biomarker levels of toxicants such as several heavy metals, acrylamide, glycideamide, and several volatile organic compounds (VOCs) compared with their white-collar counterparts. Moreover, blue-collar workers from these industries have toxicant concentrations exceeding acceptable levels: arsenic (16%–58%), lead (1%–3%), cadmium (1%–11%), glycideamide (3%–6%), and VOCs (1%–33%). Blue-collar workers have higher toxicant levels relative to their white-collar counterparts, often exceeding acceptable levels associated with noncancer effects. Our findings identify multiple occupations to prioritize for targeted interventions and health policies to monitor and reduce toxicant exposures.

## Introduction

Data from the World Health Organization suggest that exposures to hazardous chemicals in an occupational setting are responsible for over 370 000 premature deaths annually on a global scale.^1^^,^^2^ Such findings lend urgency to characterize occupational exposures to identify workers from which industries or jobs are susceptible to adverse effects from toxicant exposures. Many studies tend to focus on one chemical or one chemical family to evaluate occupational exposures.^3^^,^^4^ In doing so, these studies may miss the complete picture of being exposed to a slew of toxicants if the focus is only directed at one chemical or one chemical family. Furthermore, exposures to multiple chemicals can further increase the risk of a disease. For a few studies that have investigated across multiple chemicals, they have narrowed their focus on a limited set of industries and job titles.^5^^,^^6^ Thus, there is a need for a comprehensive, untargeted approach to study occupational exposures for a wide range of chemicals across a variety of occupations.

Furthermore, many studies on occupational exposures use estimates of exposures based on job titles or air measurements at the workplace.^3^^,^^5^^,^^6^ These indirect measures are limited in their ability to accurately quantify the distribution of chemical exposures within the human body. In contrast, human biomonitoring provides a more direct estimate of exposure while also integrating exposures which derive from multiple sources and pathways. In addition, another advantage of biomonitoring data is that it provides an internal dose that can be related to a toxicological response.^7^ In particular, biomonitoring equivalents define a concentration cutoff of a chemical or its metabolites in a biological medium such as blood, urine, or serum based on acceptable exposure values such as reference dose, tolerable daily intakes, or minimal risk levels.^8^ Several studies have used biomonitoring equivalents as a screening method to evaluate risk from exposures to environmental toxicants in the general population.^8^^,^^9^ However, few studies have used biomonitoring equivalents in an occupational context to determine prevalence of workers with concentrations above acceptable levels by industry and job description.^10^^,^^11^ Such insight will help identify which toxicants and occupations should be prioritized for further human biomonitoring, health risk evaluation, and targeted interventions.

Chemical exposures have been implicated as etiological agents in adverse effects on the nervous, reproductive, immune, and cardiovascular systems. Such toxic chemicals include heavy metals such as lead and cadmium, volatile organic compounds (VOCs), polyaromatic hydrocarbons (PAHs), phthalates, and smoking-related compounds. Several epidemiological and toxicological studies have implicated the neurotoxicity of heavy metals, especially lead^12^ and cadmium.^13^ Many studies have shown the reproductive toxicity of phthalates^14^ and PAHs.^15^ PAHs^16^ and VOCs such as toluene and benzene^17^ are also known to elicit an inflammatory response individually and in combination.^18^ Heavy metals have been recently linked to cardiovascular disease.^19^ Tobacco exposures have been causal factors in several noncancer effects such as respiratory problems,^20^ heart disease,^21^ infections,^22^ and fertility problems.^23^ Overall, the existing literature lends urgency to identify which workers from which industries and occupations are at high risk for adverse effects from chemical exposures.

Our goal is to broadly identify occupations susceptible to high exposure and risk associated with combinations of multiple toxicants. To accomplish this goal, we used data from the National Health and Nutrition Examination Survey (NHANES), which measures a broad range of 517 chemical biomarkers as part of mission of the Centers for Disease Control and Prevention (CDC) to assess the health and nutritional status of the US noninstitutionalized population. Occupational information, particularly the 21 industrial and 19 occupation codes, are also available. Our objectives are to (1) define differences in chemical exposures based on occupation description, (2) identify occupational groups with similar chemical exposure profiles, and (3) identify occupational groups with chemical biomarker levels exceeding acceptable health-based biomarker levels.

## Methods

### Study population

**Figure 1.**
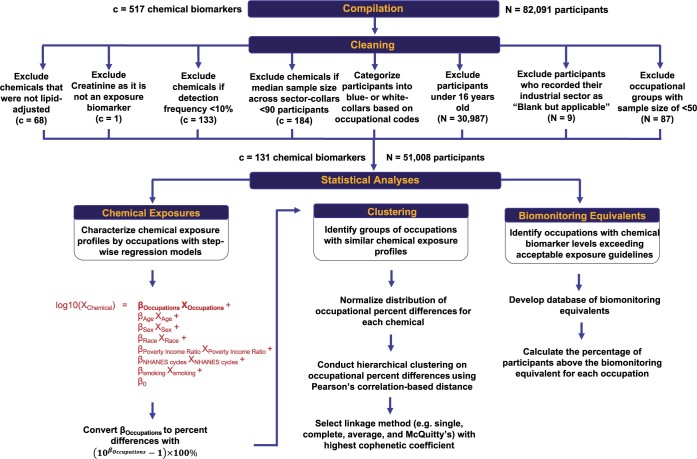
Schematic description on curation of chemical biomarker and inclusion criteria of participants and of the analytical methods used to characterize occupational variations in chemical exposures. Reference group for the analysis on the industry-collar combinations is white collars from public administration.

Since 1999, the CDC has conducted the Continuous NHANES to collect cross-sectional data on demographic, socioeconomic, dietary, and health-related information in the US population. For this analysis, we combined data from the chemical biomarker, demographic, and occupational datasets between years 1999 and 2014 for an initial sample of 82 091 participants. We categorized participants as different groups of unemployment status using the questionnaires on the type of work done last week and main reason for not working last week. We categorized the workers into their corresponding industry by using the publicly available industry code on the participants’ current job. We categorized participants into white- or blue-collar workers by using the publicly available occupational codes on the participants’ current job and the US Department of Labor definition of blue-collar.^24^ Blue-collar workers are defined as workers who perform repetitive tasks with their hands, physical skill, and energy. The industry and occupational codes can be found at https://wwwn.cdc.gov/nchs/nhanes/search/datapage.aspx?Component=Questionnaire. We tabulated the job occupation description and the collar category in Table S1. We then excluded participants under 16 years old (*N* = 30 987) as this is the minimum age at which NHANES recorded occupational status. We excluded participants who recorded their industry as “Blank but applicable” (*N* = 9). We also excluded participants (*N* = 87) from the following occupational groups if the sample size was less than 50 participants: blue-collar workers from “Armed Forces” and “Finance, Insurance” and white-collar workers from “Armed Forces,” “Private Household,” and “Mining.” These exclusion and inclusion criteria are further detailed in Figure 1. The resulting sample size of our studied population was 51 008 participants. Table S2 provides the sample size of each industry–collar combinations and unemployment.

The National Center for Health Statistics research ethics review board provided ethical approval of the study. All participants provided written informed consent.

### Chemical biomarkers of occupational exposures

We defined chemical biomarker, *c*, as an indicator of environmental exposure that can be measured in blood, serum, or urine. We replaced all measurements below the limit of detection (LOD) with the LOD divided by the square root of 2, as recommended by the CDC^25^ to produce reasonably unbiased means and standard deviations.^26^ For di-2-ethylhexyl phthalate (DEHP) and arsenic separately, we calculated the sum of metabolites to compare with respect to the biomonitoring equivalents. We used mono-(2-ethyl-5-oxohexyl) phthalate, mono-(2-ethyl-5-hydroxyhexyl) phthalate, mono-(2-ethylhexyl) phthalate, and mono-2-ethyl-5-carboxypentyl phthalate to calculate the summation of DEHP metabolites by adding the mass weights together. We calculated the summation of arsenic metabolites with monomethylarsonic acid and dimethylarsonic acid as the available biomonitoring equivalent for arsenic metabolites is the sum of these two metabolites. Therefore, we have a total of *c* = 517 chemical biomarkers.

Then, we further excluded chemicals that (a) have a median sample size of less than 90 participants across the occupational groups based on a power calculation (Table S3), (b) have non-lipid adjusted measurements when lipid adjusted measurements are available, and (c) have a detection frequency less than 10%. We chose 10% as the threshold to include more lower detected chemical biomarkers. We delineated in detail which chemical biomarkers were excluded from our analyses in Text S1. We tabulated the inclusion criteria for each chemical in Table S4. The final dataset for analysis consisted of 131 chemical biomarkers from 12 classes (Figure 1 and Table S5). We tabulated the sample size of each chemical in Table S6. We tabulated the distribution statistics for each chemical in Table S7. We displayed the detection frequency and percentages of participants with measurements by each combination of chemical biomarker and occupational group in Figures S1 and S2, respectively. Laboratory methods used to measure the chemical biomarkers are provided at https://wwwn.cdc.gov/nchs/nhanes/search/datapage.aspx?Component=Laboratory.

### Statistical analysis

We performed all analyses using R version 3·6·0. Our analytic code is publicly available on GitHub (https://github.com/vynguyen92/nhanes_occupational_exposures). We applied the survey weights to all our statistical models to (1) account for NHANES sampling designs and (2) enable the generalizability of our findings to the non-institutionalized, civilian US population.

We used multivariate regression models to evaluate differences in the chemical biomarker levels across the occupational groups, which includes the industry–collar combinations and unemployed groups. We conducted a series of stepwise linear regression models with the log_10_ transformed chemical measurements as the outcome variable and the main predictor as the occupation groups with the reference group as white collars from Public Administration. The selection of the reference group was based on the a priori hypothesis that white collar workers from Public Administration would be exposed to toxicants at relatively low levels for most toxicants. We adjusted for age (continuous), sex (categorical), race/ethnicity (categorical), poverty income ratio (continuous), NHANES cycle (continuous), and tobacco use/exposure status using serum cotinine levels (continuous). We additionally adjusted for urinary dilution by using urinary creatinine (continuous) for chemical biomarkers measured in urine. Race is self-reported by the participants. The poverty income ratio is defined by dividing the total family income by the poverty income line. A poverty income ratio lower than 1 implies that the participant’s total family income is below the poverty income line. For ease of interpretation, the regression coefficients for the occupational groups were converted to percent differences [10^coefficient^ − 1] × 100. To identify significant comparisons while maintaining a lower false positive rate, we used the false discovery rate (FDR) method on the p-values of the regression coefficients pertaining to the occupational groups.^27^

The non-random sparsity of the chemical biomarker dataset in the NHANES worker population creates challenges in applying machine-learning techniques to group individual workers together based on similarity in chemical exposure profiles. Applying most machine-learning techniques requires a complete dataset.^28^ However, as no worker has data available for all studied toxicants (Table S8 and Figure S3), we cannot characterize the chemical profile for each individual worker. Such challenges limited studies to characterizing combinations of toxicant exposures within a specific chemical family, but workers are exposed to multiple toxicants across a variety of chemical families at their workplace. Instead of characterizing the toxicant profile of a given worker, we can characterize the profile for a group of individual workers to address this sparsity issue by identifying clusters of occupations with similar chemical exposure profiles. Thus, we performed hierarchical agglomerative clustering analyses on the dataset of percent differences for the occupational groups. Text S2, Table S9, and Figure S4 describe the methodology used to identify clusters of occupations with similar chemical exposure profiles.

To identify susceptible occupations with chemical biomarker levels exceeding acceptable exposure levels, we compared workers’ biomarker levels to biomonitoring equivalents. Biomonitoring equivalents are defined as a concentration cutoff of a chemical or its metabolites in a biological medium such as blood, urine, or serum. These concentrations cutoffs correspond to acceptable or safe exposure values such as reference dose, tolerable daily intakes, or minimal risk levels.^8^ We, first, performed a literature review to develop a database of biomonitoring equivalents derived from using physiological based pharmacokinetic modeling (PBPK)^7-10^ (Tables S10 and S11). In the development of the biomonitoring equivalents, researchers used PBPK modeling to estimate blood concentrations of the chemical from exposure guideline associated with inhalation and ingestion routes.^8^ There are three types of effects for the biomonitoring equivalents: noncancer, inhalation cancer, and ingestion cancer. Noncancer effects can include mutagenicity, developmental toxicity, neurotoxicity, reproductive toxicity, immunotoxicity, and hepatotoxicity. The biomonitoring equivalents for noncancer effects are based on references doses or the equivalents. Cancer effects are specific for different route of exposures. The biomonitoring equivalent for cancer effects are based on a 1/10 000 extra risk for cancer. Then we used this database to calculate the percentage of participants above the biomonitoring equivalent for each occupation and each chemical to identify participants who have chemical biomarker levels above a safe concentration. We used hierarchical clustering to group the occupations who have similar profiles in chemical biomarker levels exceeding acceptable levels.

Text S3 provided full details on the methodology to quantify the contribution of occupation in explaining chemical biomarker levels. Text S4 provided full details on the sensitivity analyses to characterize the influence of smoking on differences in chemical biomarker levels by occupation.

### Role of the funding source

The funders of the study did not have a role in study design, data collection, data analysis, data interpretation, and writing of the manuscript. All authors have full access to the data in the study and accept responsibility for the decision to submit for publication. The corresponding author had full access to all the data and the final responsibility to submit for publication.

## Results

All figures are available on our interactive app at https://chiragjp.shinyapps.io/nhanes_occupational_exposures/.

### Study population

**Figure 2.**
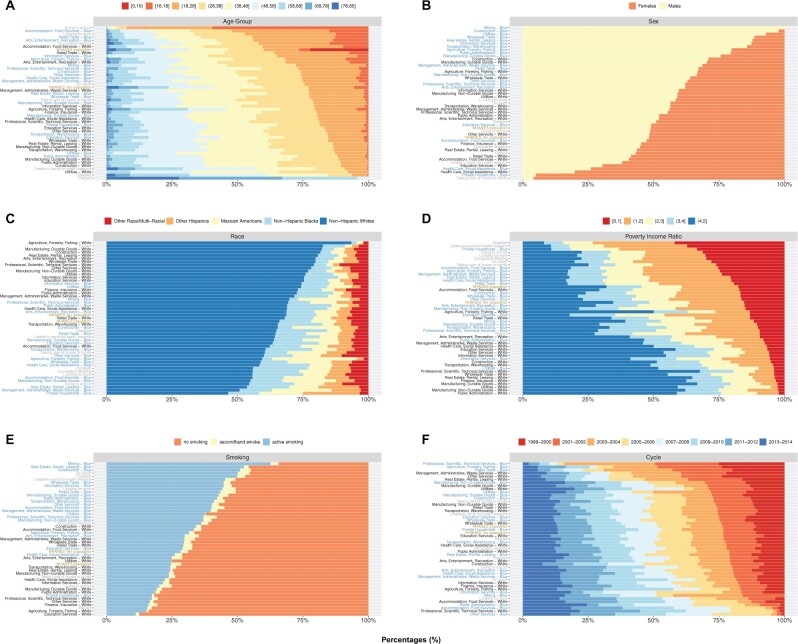
Panel of bar plots showing the percentage of participants by (**A**) age group, (**B**) sex, (**C**) race/ethnicity, (**D**) poverty income ratio (PIR), (**E**) smoking status, and (**F**) NHANES cycle for each industry–collar combination and unemployment status. The occupational groups are ordered in ascending order based on percentage of (A) participants who are 28 years and younger, (B) males, (C) Non-Hispanic Whites, (D) PIR = [0,1] (i.e. participants who are below the poverty income line), (E) participants who do not smoke, and (F) participants in 1999–2002. The “NHANES Population” consists of all participants in 1999–2014. The “NHANES 16+ Population” consists of participants in 1999–2014 and are 16 years old or older. Smoking status is defined using serum cotinine levels: no smoking ≤ 1 ng/mL, secondhand smoke 1–3 ng/mL, and active smoking > 3 ng/mL. These individual figures and text for the statistics are available on our interactive app at https://chiragjp.shinyapps.io/nhanes_occupational_exposures/ in option “Bar plot of percentages of demographic categories” under “Choose a plot.”

Tables 1 and 2 present population characteristics for the 51 008 NHANES participants from 1999 to 2014. Figure 2 shows the percentage of categories for age group, sex, race, poverty income ratio, smoking status, and study period for each occupational group. Participants working in blue-collar jobs tend to be, on average, younger compared with those working in white-collar jobs (Figure 2A). Blue-collar jobs are primarily occupied by males, while females tend to work in private household, health care, and education (Figure 2B). White-collar jobs are predominantly comprised of Non-Hispanic White participants, whereas blue-collar workers tend to be more diverse (Figure 2C). There is a socioeconomic gradient with white-collar workers having higher poverty income ratio (i.e. higher socioeconomic status) compared with blue-collar workers (Figure 2D). There is a substantial proportion of active smokers in blue collar jobs as well as those “Looking for work,” “Disabled,” or “On layoff,” whereas the proportions of active smokers are much lower in white-collar jobs (Figure 2E). There is a relative uniform distribution of participants by study period for each occupation (Figure 2F). These figures are available on our interactive app at https://chiragjp.shinyapps.io/nhanes_occupational_exposures/.

**Table 1.** Population statistics of the categorical variables for 51 008 NHANES participants who are eligible to have an occupation title

	*N* (%)
**Sex**	
Males	24 723 (48.2)
Females	26 285 (51.8)
**Race**	
Mexican	10 049 (8.28)
Other Hispanics	3712 (5.42)
Non-Hispanic Whites	22 424 (68.59)
Non-Hispanic Blacks	11 158 (11.50)
Other Race/Multi-Racial	3665 (6.21)
**Cycle**	
1999–2000 (Cycle 1)	6036 (10.93)
2001–2002 (Cycle 2)	6627 (12.53)
2003–2004 (Cycle 3)	6191 (12.10)
2005–2006 (Cycle 4)	6132 (12.38)
2007–2008 (Cycle 5)	6530 (12.65)
2009–2010 (Cycle 6)	6875 (12.86)
2011–2012 (Cycle 7)	6164 (13.14)
2013–2014 (Cycle 8)	6453 (13.41)

*Note*: NHANES sampling design is accounted in calculating percentages (%), while counts (*N*) pertain to the number of NHANES participants.

**Table 2.** Distribution statistics of the continuous variables for 51 008 NHANES participants who are eligible to have an occupation title

	*N* (%)	Minimum	5th	10th	Median	Mean (SE)	90th	99th	Maximum
Age (years)	51 008 (100)	16	17	21	44	44.7 (0.20)	71	83	85
Poverty income ratio (−)	46 441 (91.0)	0	0.49	0.77	2.86	2.93 (0.031)	5	5	5
Serum cotinine (ng/mL)	45 376 (88.9)	0.011	0.011	0.011	0.061	58.55 (1.56)	250	514	1820
Urinary creatinine (mg/dL)	47 357 (92.8)	0	24	35	113	126.48 (0.82)	235	374	882

*Note*: NHANES sampling design is accounted in the calculations of the distribution statistics, while counts (*N*) pertain to the number of NHANES participants.

### Differences in chemical biomarker levels by occupational groups

**Figure 3.**
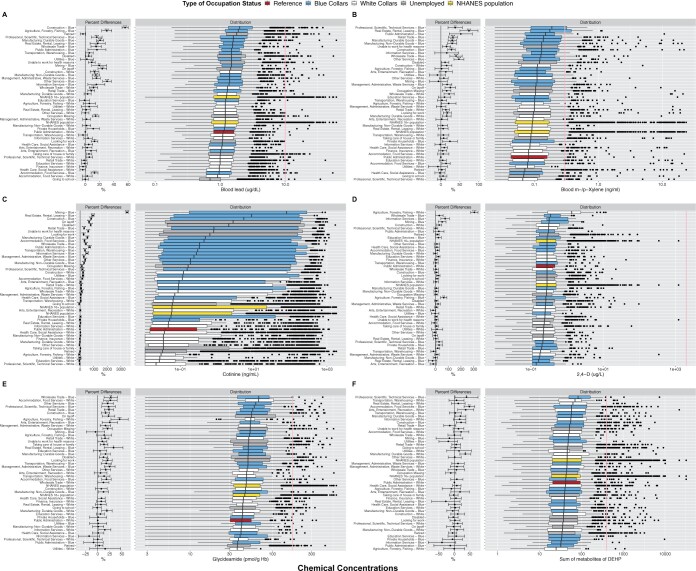
Panel of boxplots of chemical distribution for (**A**) blood lead, (**B**) m-/p-xylene, (**C**) cotinine, (**D**) 2,4-d, (**E**) glycidamide, and (**F**) sum of DEHP metabolites. The pink line represents the biomonitoring equivalent of the chemical for noncancer effects. The “NHANES Population” consists of participants in 1999–2014. The “NHANES 16+ Population” consists of participants in 1999–2014 and are 16 years old or older. Percent differences are derived from fully adjusted models, which were adjusted for age, sex, race/ethnicity, poverty income ratio, study period, and serum cotinine (biomarker of smoking). Reference group for the occupational groups is comprised of white collars from Public Administration. Number of asterisks indicate statistical significance of the percent differences: *(*P*-value ∈ ([0.01, 0.05]), **(*P*-value ∈ ([0.001, 0.01]), and ***(*P*-value ≤ 0.001). The *P*-values corrected for multiple comparison with the Benjamini and Hochberg FDR procedure of 5%. These individual figures and text for the statistics are available on our interactive app at https://chiragjp.shinyapps.io/nhanes_occupational_exposures/ in option “Box and forest plots of differences in chemical concentrations” under “Choose a plot.”

Figure 3 displays the differences in chemical biomarker levels across the occupational groups using regression and distribution statistics for the following chemicals: lead, m-/p-xylene, cotinine, 2,4-d, glycidamide, and sum of DEHP metabolites. We selected these chemicals for at least one of the following reasons: (1) availability of having a biomonitoring equivalent and (2) existence of statistically significant differences in biomarker levels across the occupational groups. Biomonitoring equivalents are available for the selected chemicals except for cotinine. In Figure 3A, blood lead is, on average, significantly higher in blue-collar workers from several industries and unemployed groups such as “Looking for work” (12.88%, *P*-value < 0.001), “On layoff” (23.86%, *P*-value < 0.001), and “Disabled” (4.8%, *P*-value = 0.034) compared with the reference group of white-collar workers from “Public Administration.” We observed a similar pattern in blood cadmium (Figure S5). In contrast, metabolites of mercury display the opposite exposure patterns to those of lead and cadmium. Total blood mercury levels of most blue-collar workers are substantially and significantly lower compared with those of white-collar workers (Figure S6). Similarly, m-/p-xylene (Figure 3B) and toluene (Figure S7) are higher in blue-collars and unemployed participants in “On layoff,” “Disabled,” and “Unable to work for health reasons” compared with white-collar workers. Similar results are observed for several PAHs such as 1-pyrene, 2-fluorene, 3-fluorene, and 1-naphthol (Figure S8), but the signals are not as strong as those for toluene and m-/p-xylene. It is noteworthy that within the same industry such as “Professional, Scientific, Technical Services,” levels of m-/p-xylene are substantially different between white versus blue collars. Figure 3C shows a smoking gradient with blue-collar workers and unemployed participants having substantially higher levels of cotinine compared with white collars and the NHANES populations. NNAL, which is primarily found in tobacco products, shows a similar trend (Figure S9). The signals for the smoking-related compounds are among the strongest and most substantial. Figure 3D shows how concentrations of an herbicide, 2,4-d, are significantly and substantially higher in blue-collar and white-collar workers from “Agriculture, Forestry, Fishing” along with blue-collar workers from “Information Services.” Although none of the participants have 2,4-d levels exceeding the guideline biomonitoring equivalent levels. DEET acid, a metabolite of DEET and a common ingredient used in insect repellant, shows a similar pattern (Figure S10). In Figure 3E, glycidamide levels, on average, are significantly higher in blue collars from “Wholesale Trade,” “Other Services,” “Retail Trade,” “Construction,” “Arts, Entertainment, Recreation,” “Real Estate, Rental, Leasing,” “Transportation, Warehousing,” “Management, Administrative, Waste Services,” and “Accommodation, Food Services.” In addition, glycidamide levels are also significantly higher in white collars from “Accommodation, Food Services,” “Agriculture, Forestry, Fishing,” “Other Service,” and “Manufacturing: Non-Durable Goods.” Acrylamide shows similar pattern (Figure S11). These two chemicals are found in food prepared at high temperature via frying, baking, or roasting and are used in textile, paper processing, and cosmetics. In Figure 3F, levels of the sum of urinary DEHP metabolites are significantly higher in “Professional, Scientific, Technical Services” and “Accommodation, Food Services” compared with the reference group. Phthalates, in general, are used as plasticizers. On the contrary, mono-ethyl phthalate, an indicator of personal care product usage, are, on average higher, in the reference group (Figure S12).

### Chemical exposure profiles

**Figure 4.**
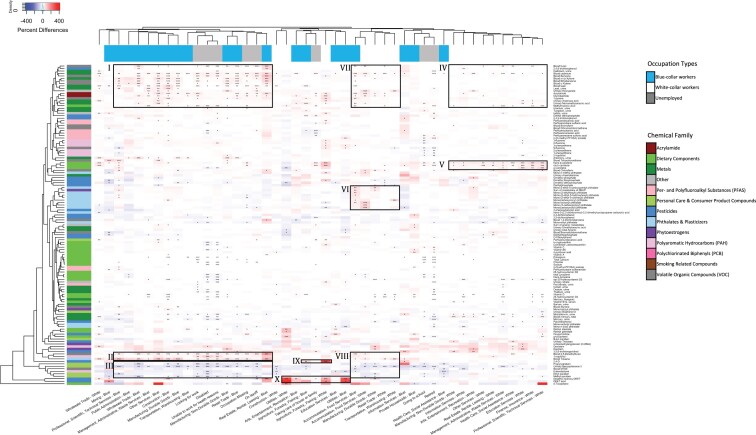
Heatmap of percent differences in chemical biomarker concentrations by occupational group, relative to white collars from Public Administration. Chemical biomarkers in white color indicate that the concentrations are the same between the given industry–collar combination and the reference group. The color bar for the columns represents the collar categorization and unemployment. The color bar for the rows represents the chemical classes. Blue presents the blue-collar workers. White represents the white-collar workers. Gray presents the unemployed participants. The dendrogram of the occupational groups is defined based on using the average linkage function with Pearson’s correlation-based distance. Results are adjusted for age, sex, race/ethnicity, poverty income ratio, study period, and serum cotinine (biomarker of smoking). Number of asterisks indicate statistical significance of the percent differences: *(*P*-value ∈ ([0.01, 0.05]), **(*P*-value ∈ ([0.001, 0.01]), and ***(*P*-value ≤ 0.001). This figure and text for statistics are available on our interactive app at https://chiragjp.shinyapps.io/nhanes_occupational_exposures/ in option “Heatmap of differences in chemical concentration” under “Choose a plot.”

Figure 4 shows differences in chemical exposure profiles by occupational groups for the 131 studied chemicals. The chemical exposure profiles of blue-collar workers are more similar to each other and those of unemployed participants than to their white-collar counterparts. For example, blue-collar workers from “Construction,” “Other Services,” “Professional, Scientific, Technical Services,” “Real Estate, Rental, Leasing,” “Manufacturing,” and “Wholesale Trade” have some of the highest biomarker levels of heavy metals, such as cadmium and lead, PAHs, and volatile organic chemicals (VOCs), including m-/p-xylene and toluene (Zones I and II), but have lower levels of metabolites of chemicals used in personal care products such as BP-3, a UV blocking chemical used in sunscreen, and the parabens (Zone III). Interestingly, participants who are “Looking for work,” “On layoff,” “Disabled,” “Unable to work for health reasons,” and “Occupation Missing” have similar chemical exposure profiles to the aforementioned blue-collar workers (Zones I–III). The far right of the heatmap consists of mostly white-collar workers with their chemical exposure profile described by lower levels of heavy metals and VOCs (Zone IV) but higher levels of dietary components such as orange or red plant pigments found in fruits and vegetables such as trans-b-carotene, cis-b-carotene, and a-carotene (Zone V). These occupational groups have the most similar chemical exposure profiles to that of the reference group of white-collar workers from “Public Administration” as the percent differences across most studied chemicals are near 0, that is, the blue and red boxes are faded. Within the food services cluster, which includes blue and white collars from “Transportation, Warehousing” and “Accommodation, Food Services,” the phthalates signal is particularly stronger in the blue-collar workers from “Accommodation, Food Services” (Zone VI). For the food service clusters, biomarker levels of heavy metals and some VOCs (Zone VII) are lower compared with those for the blue-collar workers and unemployed participants in Zone I. However, biomarker levels for VOCs and chemicals used in personal care and consumer products (Zone VIII) are on par with those observed in Zones II and III. In terms of pesticide exposures, both blue- and white-collar workers from “Agriculture, Forestry, Fishing” show some of the highest levels of 2,4-d (Zone IX), while only white-collar workers from the same industry show higher levels of DEET metabolites (Zone X). In addition, both blue- and white-collar workers from “Utilities” have some of the highest levels of DEET metabolites (Zone X). We excluded smoking-related compounds such as NNAL and cotinine from the figure, or else we would not be able to observe any signals from the other chemical biomarkers (Figure S13).

### Occupational groups with chemical biomarker levels exceeding acceptable guidelines

**Figure 5.**
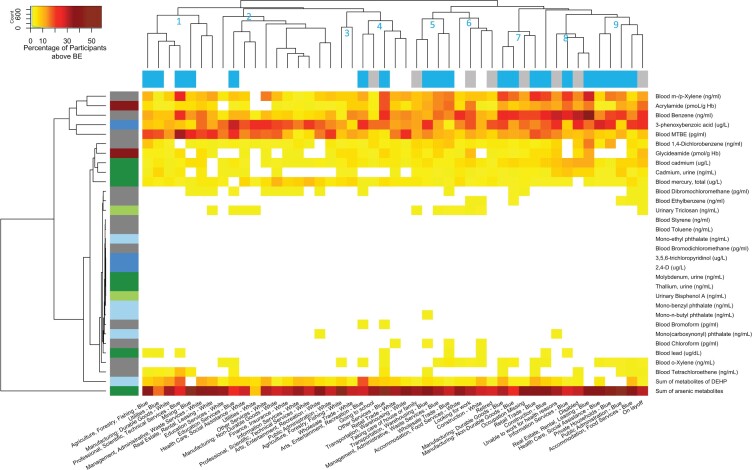
Heatmap of percentages of workers with biomarker levels exceeding biomonitoring equivalents for noncancer effects. The numbers in blue text on the dendrogram indicate the clusters of occupational groups. Chemical biomarkers in white color indicate that no worker in a given occupational group has biomarker levels exceeding acceptable guidelines. The color bar for the columns represents the collar categorization and unemployment. Blue presents the blue-collar workers. White represents the white-collar workers. Gray presents the unemployed participants. This figure and text for statistics are available on our interactive app at https://chiragjp.shinyapps.io/nhanes_occupational_exposures/ in option “Heatmap of percentages of workers above biomonitoring equivalents” under “Choose a plot.”

Figure 5 shows the percentage of workers with biomarker levels exceeding the biomonitoring equivalents for a given chemical biomarker and a given occupational group. The biomonitoring equivalents are for non-cancer effects. Figure S14 is a dendrogram that displays approximately unbiased (AU) *P*-values and bootstrap probability (BP) values to show how the clusters are supported by the data. Our hierarchical clustering analysis on the occupational groups shows a variety of clusters: two with predominantly white-collar workers (Clusters 2 and 3), four with blue-collar workers and unemployed participants (Clusters 5, 7, 8, and 9), one with blue- and white-collar workers (Cluster 1), one with white-collar workers and unemployed participants (Cluster 6), and one with all three types of workers (Cluster 4). The two white-collar clusters (Clusters 2 and 3) and four blue-collar and unemployed clusters (Clusters 5, 7, 8, and 9) suggest that blue collars and unemployed groups have similar chemical risk profiles to each other, and such profiles are different from the chemical risk profiles of the white collars. Several blue collar jobs along with unemployed groups (Clusters 5, 7, 8, and 9) such as those who are “On layoff,” “Unable to work for health reasons,” and “Disabled” have some of the highest percentages of participants with biomarker levels exceeding acceptable health levels for VOCs such as m-/p-xylene, benzene, pesticides such as 3-phenoxybenzoic acid, heavy metals such as cadmium, lead, and arsenic, metabolites of DEHP, and acrylamide and its metabolite glycideamide. The other mixed clusters (Clusters 1, 4, and 6) displayed a more similar chemical risk profile as the clusters of blue-collar workers and unemployed participants (Clusters 5, 7, 8, and 9) than those of the white-collar clusters (Clusters 2 and 4). These findings suggest that toxicants exceeding safety concentrations should be further monitored to understand why biomarker levels are exceeding acceptable guidelines in these occupational groups. It worth noting that white-collar workers within the mixed clusters (Clusters 1, 4, and 6) displayed elevated chemical risk compared with the white-collar workers who are in the predominantly white-collar clusters (Clusters 2 and 4). This finding suggests that the industry of the white-collar workers in the mixed clusters may be more informative of a worker’s exposure and subsequential risk than just the collar category alone. Figures S15 and S16 show the percentage of participants with excessive toxicant levels for the cancer effects.

## Discussion

In this study, we systematically characterize differences in chemical biomarker levels across a diverse suite of chemical contaminants and occupations. This is the first application of hierarchical clustering on differences by chemical exposures to identify groups of workers with similar chemical exposure profiles. This is also the first study to determine the percentage of a given occupation who are exceeding acceptable levels for a broad set of toxicants. Furthermore, this is the first application of hierarchical clustering to systematically identify which chemicals for which occupations have biomarker levels exceeding acceptable guidelines. Our findings are informative for identifying which workers are susceptible to higher exposures from which toxicants.

Contact with products and equipment may explain higher biomarker levels of heavy metals such as lead and cadmium, PAHs, and VOCs such as toluene and benzene found in blue-collar workers and pesticides found in workers from utilities, forestry, agriculture, and fishing. In addition, such contact with products and equipment may also explain why several blue-collar workers have similar chemical exposure profiles. Higher lead levels found in blue collars may be due to the presence of lead in old and commercial paint, car parts, batteries, glass, and consumer products made of plastics.^29^ In addition, this same group of workers may be exposed to cadmium via industrial uses of cadmium in making batteries, plating, pigments, and plastics.^30^ Sources of occupational PAH exposures to this group may be due to engaging in tasks that involve combustion emission.^31^ Similarly, higher VOCs levels may also be due to working with products containing VOCs.^32^ Higher biomarker levels of herbicides were expected to be found in workers from agriculture and forestry industries as they use herbicides to control for undesirable vegetation.^33^^,^^34^ Workers from the fishing industry also have elevated concentrations levels of herbicides, but it is unlikely that they use herbicides due to their concerns of how chemicals are polluting the waters, killing animals, and disrupting their way of life.^35^ A likely explanation may be due to frequent contact with vegetation sprayed with herbicides, and hence, elevated herbicides exposures may be indicative of fishermen being outdoors. In addition, workers from agriculture, forestry, and fishing industries were also expected to have elevated levels of DEET, an active ingredient found in insect repellant products, as they apply insect repellants to prevent contacting insect-borne diseases when being outdoors.^36^^,^^37^ Surprisingly, we observed that workers from the utilities industry have elevated levels of DEET. The utilities industry provides services such as electric power, natural gas, steam supply, water supply, and sewage removal,^38^ so why would utilities workers use insect repellant products and/or be concerned with insect-borne disease? This may be explained by how such services required utilities workers to be outside. Workers in the food and/or accommodation services were not grouped with majority of the blue-collar workers even though they have similar biomarker concentrations of the aforementioned toxicants, albeit the concentrations are slightly lower. Worker in the food and/or accommodation services have the highest levels of phthalates, which are found in packaging^39^^,^^40^ and flooring materials.^41^ Such elevated levels of phthalates may explain how workers from food and/or accommodation services are separate from the clusters of the blue-collar workers, even though the exposure profiles for many other toxicants are similar. As white-collar workers have little to no contact with such products and equipment, their biomarker levels for such toxicants are lower than those of blue-collar workers and workers in food and/or accommodation services. Thus, such substantial differences in chemical biomarker levels led to how majority of white-collar workers form their own cluster. Overall, higher biomarker levels of heavy metals, PAHs, and VOCs in predominantly blue-collar workers and phthalate levels in workers from food and/or accommodation services may be due to contact with products containing these chemicals. This suggests that the forementioned workers should be further examined to understand sources of exposures for such toxicants and reasons for exceeding acceptable levels.

On the other hand, behaviors associated with higher socioeconomic status may explain why most white-collar workers have higher levels of mercury along with chemicals used in personal care products such as parabens and BP-3, a biomarker of sunscreen use. While mercury^42^ is used in many industries, it is less likely that higher biomarker levels of arsenic metabolites in white collars are due to occupational exposures. Although health care workers may be exposed to mercury via medical or dental equipment.^43^ Higher mercury biomarker levels among these white-collar workers may indicate higher fish consumption,^44^ which is associated with higher socioeconomic status instead of an indicator of occupational exposures.^45^ It is also doubtful that white-collar workers are manufacturing products containing parabens and BP-3.^46^^,^^47^ Instead, as BP-3 and parabens are widely used in personal care products,^48^ higher levels of these chemicals may suggest that these workers are using more cosmetic products, which can have a major role in strategic self-presentation.^49^ Higher levels of BP-3 in white-collar workers may likely result from how majority of white-collars are Non-Hispanic Whites.^50^ People with lighter skin pigmentation tends to use more sunscreen and/or products containing BP-3.^51^ Overall, elevated levels of mercury, dietary factors, and BP-3 found white-collar workers likely indicate behaviors associated with socioeconomic status.

Many studies using NHANES have been limited to studying chemical co-exposures in one chemical family due to the non-random sparsity of the chemical biomarker data.^52^^,^^53^ To address this sparsity challenge, we conducted clustering analysis on exposure differences among the occupational groups, that is, clustering analysis on statistics of the biomarker data instead of on the raw data. Our framework enabled the identification of co-exposure across a wide range of chemicals not only limited to one chemical family. This framework can be applied in other settings to help cluster observations based on similar profiles especially in a non-randomly sparse dataset. This can be done without having to form a complete dataset or impute the missing values.

Our findings lend urgency to understand how one’s occupation can be a route for smoking initiation in young people and consequentially exposure to other toxicants. The connection between being a blue collar, active smoker, and younger may suggest that being an active smoker is part of the culture of a blue-collar worker.^54^ Furthermore, blue-collar workers are additionally exposed to VOCs, PAHs, and heavy metals, since these chemicals have been detected in tobacco products. Moreover, our sensitivity analysis points to how large variations in biomarker levels for VOCs, PAHs, and heavy metals are explained by tobacco use. Our findings suggest that participants, particularly blue-collar workers, may be exposed to such toxicants via smoking. Interestingly, blue-collar workers have higher levels of nicotine metabolites and dietary components such as beta- and alpha-carotenes. This finding is especially alarming, since cancer-preventative trials have shown that vitamin A analogs, alone or in combination with Vitamin E, are risk factors for lung cancer and mortality in active smokers.^55^^,^^56^ Our findings can inspire future studies to develop interventions to understand cultural and behavioral factors leading to smoking initialization and implement evidence-based regulations on tobacco control to prevent the younger population from initiating smoking.^57^

Environmental injustice is defined as the disproportionate exposures of toxicants and dietary factors and their consequential effects on health to disadvantaged groups such as individuals from racial minorities and/or low socioeconomic status.^58^ Blue-collar workers are disproportionally exposed to some of the most toxic chemicals with several found at concentration exceeding acceptable guidelines for cancer and noncancer effects.^59^^,^^60^ Furthermore, many blue-collar workers may come from the lowest socioeconomic status.^61^ We observed that occupation alone can explain up to 14% and 3%–7% of the variation of the biomarker levels of some toxicant and dietary factors, respectively, but is up 5% and 2%–3% when adjusting for covariates such as age, sex, race, poverty income ratio, study period, and smoking. While occupation remains an important determinant of exposures independent of other confounders, the substantial decrease in variation explained suggest the intersectionality of occupation on exposures and consequential health effects. Therefore, future studies can use other statistical tools that account for the correlation among occupation and covariates. Moreover, future studies can also use machine-learning feature selection methods to identify the most important predictors of chemical and dietary exposures to help quantify the importance of occupation in explaining biomarker levels compared with other variables. Overall, our findings call for an increase in exposure surveillance and industrial controls, effect regulations on chemical exposures, inform remediation strategies, and help implement interventions programs to improve the health of workers susceptible to toxicant exposures. Moreover, health providers such as occupational physicians and industrial health personnel can use our findings to inform their patients on preventatives measures to avoid occupations and toxicant exposures that may increase their personal and familial disease risk.

The present study has several limitations. First, as NHANES is a cross-sectional survey, we cannot make claims on causal factors of chemical exposures. Second, a limitation of using chemical biomarker data is that a delay between the time of exposure and time of data collection may prevent the detection of higher occupational exposures. This limitation is especially salient for short-lived chemical biomarkers such as VOCs, which have short half-life ranging from 2 to 128 h,^62^^,^^63^ which implies that substantially higher biomarker levels could be observed at workplace. Third, while we identified differences in chemical biomarker levels by occupation, we cannot claim that such differences are due to occupational exposures, since we do not know the source of all exposures for each study participant. Furthermore, there may be variation in exposures within the same occupational group due to how an occupational group can encompass many different types of duties and locations with varied exposures sources. Nevertheless, our findings can inspire future studies to prioritize chemicals and susceptible occupations in specific industries to measure at the workplace and/or characterize other sources of elevated biomarker levels by integrating biomonitoring with exposure monitoring. Fourth, differences in data sparsity between the occupational groups can increase the uncertainty of the occupational differences in chemical biomarker levels. By chemical, sparsity does not differ substantially for most of the occupational groups. However, the exceptions include blue-collar workers from “Retail Trade,” “Agriculture, Forestry, Fishing,” and “Professional, Scientific, Technical Services” for DEET and its metabolites, the parabens, methylmalonic acid, furan, a few phthalates, NNAL, and Vitamin D derivatives. For these mentioned chemicals, these aforementioned occupations have higher missingness compared with other occupations and have higher confidence intervals on the occupational differences. Hence, there is a need to sample for more participants for these toxicants and occupational groups. Fifth, while NHANES has biomonitoring data on 517 chemicals, we only obtained biomonitoring equivalents for 106 chemicals (20.5%). We were limited to these biomonitoring equivalents, since we selected those that were derived from the same methodology of using PBPK modeling to estimate blood concentrations from exposure guidelines. Future works can implement a more systematic approach such as an in vitro–in vivo extrapolation (IVIVE) method to expand the number of available biomonitoring equivalents.^64^ Sixth, there is potential uncertainty in how the biomonitoring equivalents are converted from exposures values to biomarker concentrations. Such uncertainty can either decrease or increase the value of the biomonitoring equivalent and in turn change the interpretation of which chemicals for which occupations should be further monitored. Moreover, biomonitoring equivalents can change due to updates based on improvements in scientific knowledge. Seventh, we made inferences on health risk from one-time chemical measurements, but a snapshot measurement may not be fully representative of long-term exposures nor subsequent potential health risk. This limitation is particularly salient for short-lived chemicals such as VOCs. Because VOCs are quickly eliminated from the body, measurements of these chemicals represent exposures at the time of collection and do not represent long-term exposure. Eighth, we did not account for how demographic characteristics (e.g. age, sex, race/ethnicity, and socioeconomic status) are strongly related to occupation. Including such characteristics as covariates in the model can inflate the differences in chemical biomarker levels across the occupation due to the multicollinearity among the covariates and occupation. Thus, future studies can use other statistical tools that account for the correlation structure of the occupation and demographic factors.

## Conclusions

Evaluating differences in chemical exposures by occupation is essential to identify occupations susceptible to higher exposures to toxicants as well as understand how occupational exposures play a role in adverse health outcomes. We applied an unbiased approach to screen across 131 chemical biomarkers to characterize the chemical exposure profiles across white- and blue-collar workers from 20 different industries and 7 unemployed groups. We developed a framework using hierarchical clustering on differences of chemical biomarker levels to identify clusters of occupations with similar chemical exposures. Our framework enabled (1) comprehensive characterization of chemical exposures across a wide variety of occupations and toxicants and (2) identification of occupations susceptible to high toxicant exposure and exceeding acceptable health-based levels. These findings can guide efforts to design targeted interventions to reduce and prevent exposures in susceptible occupations and help mitigate negative effects from toxicant exposures.

## CRediT authorship contribution statement

V.K.N.: conceptualization, data curation, formal analysis, software, visualization, writing—original draft and review and editing, and verification of underlying data. J.C.: conceptualization, funding acquisition, resources, writing—original draft and review and editing. C.J.P.: funding acquisition, resources, writing—original draft and review and editing. M.S.: writing—original draft and review and editing. O.J.: conceptualization, resources, writing—original draft and review and editing.

## Supplementary Material

osac004_Supplementary_Data
